# A pothole-filling strategy for selective targeting of rCUG-repeats associated with myotonic dystrophy type 1

**DOI:** 10.1073/pnas.2507065123

**Published:** 2026-01-09

**Authors:** J. Dinithi R. Perera, Shivaji A. Thadke, Savani W. Thrikawala, Isha Dhami, V. M. Hridya, Arnab Mukherjee, Ananya Paul, W. David Wilson, Keith W. R. Tan, Nicholas Z. W. Chan, Anh Tuân Phan, Danith H. Ly

**Affiliations:** ^a^Department of Chemistry and Institute for Biomolecular Design and Discovery, Carnegie Mellon University, Pittsburgh, PA 15213; ^b^Department of Chemistry, Indian Institute of Science Education and Research, Pune, Maharashtra 411008, India; ^c^Department of Chemistry, Georgia State University, Atlanta, GA 30302; ^d^School of Physical and Mathematical Sciences, Nanyang Technological University, Singapore 637371, Singapore

**Keywords:** DM1, Janus bases, bifacial nucleic acid recognition, cooperativity, ligands

## Abstract

We present an alternative approach to conventional small-molecule and antisense strategies for selectively targeting expanded CUG-RNA repeats associated with Myotonic Dystrophy type 1. Our alternatively designed nucleic acid ligands uniquely integrate advantageous features from both existing methods: They are compact (only three units in length), structurally resembling small molecules, yet recognize RNA targets through directional hydrogen-bonding similar to antisense oligonucleotides. Notably, these ligands exhibit greater specificity and selectivity than either approach alone. This enhanced specificity results from their bifacial recognition mechanism, wherein mismatches on one binding interface are reciprocally mirrored on the complementary face. Additionally, their short length significantly amplifies specificity, as even a single mismatch substantially reduces the overall binding free energy, effectively minimizing off-target interactions. Unlike conventional oligonucleotides, these ligands avoid binding single-stranded RNA and only recognize defined hairpin motifs via a “pothole-filling” mechanism. This method amplifies recognition specificity and selectivity, circumventing the thermodynamic penalties associated with RNA unfolding. This proof-of-concept study thus lays a foundation for developing versatile nucleic acid ligands capable of selectively targeting not only pathogenic CUG-RNA repeats in Myotonic Dystrophy type 1 but also other disease-associated triplet-repeat expansions prevalent in various neuromuscular disorders.

## Significance

This work presents a class of triplet nucleic acid ligands that combine the targeting precision of antisense oligonucleotides with the compactness of small molecules to selectively engage expanded CUG-RNA repeats implicated in Myotonic Dystrophy type 1. Featuring bifacial (Janus) recognition elements and a conformationally preorganized gamma peptide nucleic acid backbone, these ligands exhibit unusually high binding cooperativity, specificity, and selectivity for pathogenic over normal-length repeats. While cellular uptake remains an area for improvement, the design shows broad potential for targeting both RNA triplet-repeat expansions and other native RNA structures with essential physiological functions.

Myotonic dystrophy type 1 (DM1) is the most common adult-onset muscular dystrophy, affecting 1 in 8,000 individuals worldwide, with no effective treatment (1). This autosomal dominant disorder is characterized by progressive muscle wasting and cognitive impairment, caused by a CTG-repeat expansion in the 3′-untranslated region (3′-UTR) of the *DMPK* gene (2, 3), where the normal range of 5 to 35 repeats expands to 80→5,000 in affected individuals. The primary driver of DM1 is RNA toxic gain-of-function, where transcription of the expanded allele produces long CUG-RNA repeats (rCUG^exp^) that form imperfect hairpin structures (4–6), sequestering muscleblind-like protein 1 (MBNL1) and other RNA splicing regulators (2). This entrapment disrupts RNA processing and leads to widespread splicing defects (7), which are central to disease pathology. Although reduced *DMPK* expression contributes to DM1, MBNL1 sequestration plays a greater role in symptom manifestation (3). Other factors, such as protein kinase C activation (8) and repeat-associated non-ATG (RAN) translation (9), may also contribute. Fundamentally, DM1 is a disorder of dysregulated RNA splicing, driven by the aberrant binding of expanded rCUG^exp^ to MBNL1 and related proteins, resulting in their functional loss (10).

Several strategies have been explored to develop therapeutic interventions for DM1, including gene editing (11), transcriptional (12) and translational regulation (13), RNA degradation (14), and modulation of RNA–protein interactions (15). Notably, the latter approach has shown promise, with research groups such as Disney (16, 17), Zimmerman (18), and Berglund (19, 20) demonstrating that small-molecule ligands can be designed to bind rCUG^exp^ and disrupt their interaction with MBNL1. Several lead compounds have successfully restored MBNL1 function and corrected misspliced RNAs in both cell cultures and animal models and are in clinical trials (21). Similarly, targeting rCUG^exp^ with antisense oligonucleotides, specifically morpholino and gapmer variants with 2’-O-methoxyethyl modifications, Thornton (13, 22) demonstrated that DM1 phenotypes could be reversed in a mouse model. Together, these studies establish rCUG^exp^ as a molecular driver of and a bona fide therapeutic target for DM1.

Despite recent progress (15, 23), RNA targeting via small molecules and antisense oligonucleotides continues to face significant hurdles. Small molecules, while therapeutically promising, are challenging to rationally design due to RNA’s lack of defined binding pockets, resulting in reliance on screening methods, extensive optimization, and difficulties achieving specificity and selectivity (24). Antisense oligonucleotides offer programmable target engagement but suffer from poor pharmacokinetics due to their large molecular size (25). Additionally, targeting structured RNA involves overcoming substantial thermodynamic barriers, complicating effective binding. Prioritizing affinity alone may enhance binding efficiency but often compromises specificity and selectivity, leading to off-target effects and increased cytotoxicity. These limitations highlight the need for alternative strategies that simultaneously enhance RNA-targeting precision, maintain specificity and selectivity, and provide synthetic versatility, advancing the development of effective RNA-based therapeutics.

We introduce a ligand design strategy that integrates advantageous aspects of small-molecule and antisense approaches—compact size combined with high specificity and selectivity—to effectively target rCUG^exp^. Utilizing short, three-unit bifacial nucleic acid ligands as a proof-of-concept, these ligands structurally resemble small molecules yet achieve precise RNA recognition through directional hydrogen-bonding. Central to this design are bifacial (Janus) bases and conformationally preorganized gamma peptide nucleic acid (γPNA) backbones, which collectively enhance hydrogen-bonding, base-stacking interactions, cooperativity, and binding efficiency while minimizing entropic penalties. The bifacial architecture further improves specificity by ensuring reciprocal mirroring of mismatches, significantly reducing off-target binding, and enhancing selectivity by preferentially recognizing structured double-stranded RNA motifs over single-stranded regions. Unlike antisense oligonucleotides, these ligands employ a “pothole-filling” mechanism, recognizing RNA in its native secondary and tertiary conformations, thus bypassing the substantial thermodynamic barriers and kinetic inhibition associated with RNA unfolding. This approach provides a powerful and versatile solution for precise RNA targeting, overcoming critical limitations inherent to traditional antisense and small-molecule strategies.

## Results and Discussion

### Rationale.

This study is part of a broader initiative aimed at developing a comprehensive set of 16 Janus bases capable of recognizing all possible RNA base-pair combinations, including canonical and noncanonical pairs. Each Janus base is strategically optimized for uniformity in size, shape, chemical functionality, and tautomeric stability, ensuring modularity and precise hydrogen-bonding with complementary RNA bases. rCUG-repeats were selected as a model system due to their well-characterized hairpin structure formed by repeating (rCUG/rGUC)-units. These structures exhibit reduced strand-invasion barriers facilitated by internal U<>U mismatches, making them particularly suited to demonstrate the effectiveness of this bifacial recognition strategy.

**Fig. 1.**
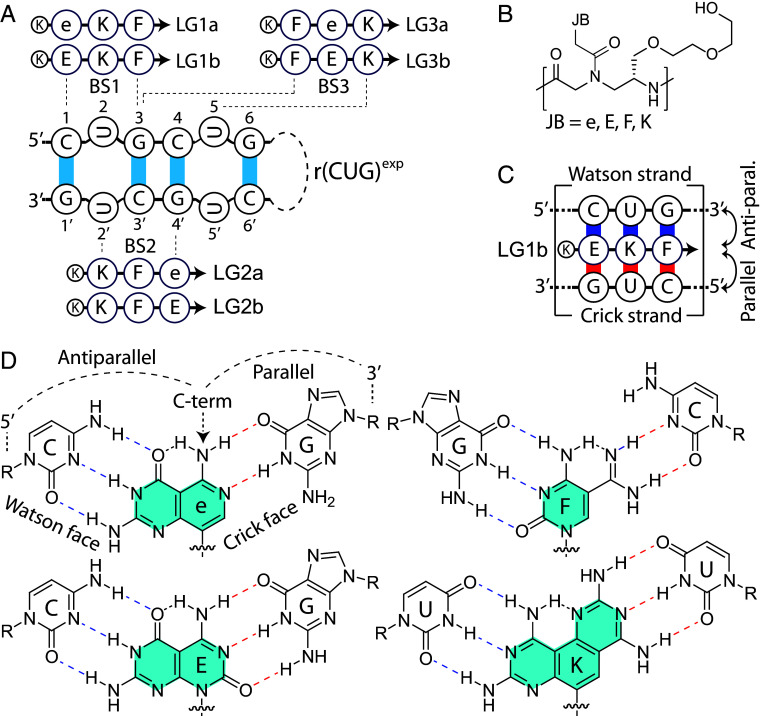
Ligand design. (*A*) Illustration of the rCUG-repeat hairpin structure, highlighting three potential ligand binding sites (BS1, BS2, BS3) and their corresponding ligands. Ligand series a and b feature three JB recognition elements (e or E, F, and K). e (first-generation) forms five hydrogen bonds with the C-G base-pair, while E (second-generation) forms six. The arrow indicates the ligand’s N terminus, and K represents *L*-lysine. (*B*) Chemical structure of the conformationally preorganized, right-handed, MiniPEG-containing gamma peptide nucleic acid (RH-MPγPNA), with JBs covalently attached. (*C*) Representative binding mode of LG1b with the cognate RNA hairpin. The Watson face (blue) is antiparallel, with the RNA 5′-end facing the ligand’s C-terminus, while the Crick face (red) is parallel, with the RNA 3′-end facing the ligand’s C-terminus. (*D*) Depiction of hydrogen-bonding interactions between JBs and designated RNA base-pairs.

This base-paired triplet-repeat RNA target offers an ideal opportunity to evaluate short, three-unit nucleic acid ligands, leveraging enhanced hydrogen-bonding, improved intra- and intermolecular base-stacking, and an entropically favorable, conformationally preorganized γPNA backbone (26, 27). As illustrated in Fig. 1*A*, these triplet ligands are strategically designed to target rCUG-repeats by shifting recognition sites in increments of one repeat unit. The first-generation Janus base (e) in “Series a” forms five hydrogen bonds with RNA C–G pairs (28, 29), while the second-generation base (E) in “Series b” establishes six hydrogen bonds, further increasing binding affinity and specificity. These Janus bases (e, E, F, and K) are incorporated into the γPNA backbone (Fig. 1*B*), and their binding orientation, binding mode, and hydrogen-bonding patterns are detailed in Fig. 1 *C* and *D*. Altogether, this study establishes a rational design framework for precise RNA targeting, significantly advancing fundamental understanding and offering potential insights for future applications.

### Molecular Modeling.

**Fig. 2.**
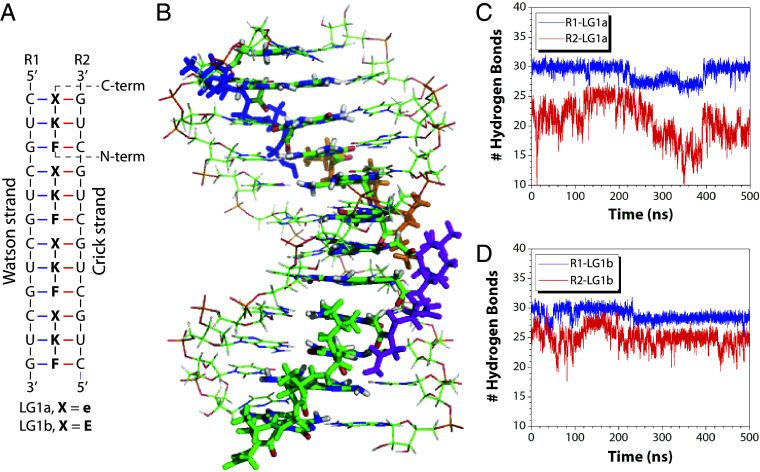
Molecular dynamics simulations. (*A*) Illustration of ligand–RNA complexes, showing four consecutively bound LG1a (X = e) and LG1b (X = E) ligands. For clarity, the C-terminal lysine residue is omitted. (*B*) Optimized structure of the LG1b–RNA complex, with four consecutively bound ligands represented in different colors. (*C* and *D*) Comparison of hydrogen-bonds formed between LG1a and LG1b with the Watson strand (R1, blue) and Crick strand (R2, red) of the RNA duplex over 500 ns simulations.

We performed molecular dynamics simulations to evaluate ligand design and hydrogen-bonding interactions between Janus bases and RNA base-pairs, using ligands LG1a and LG1b bound to an RNA duplex containing four consecutive binding sites (Fig. 2*A*). Systems were constructed, solvated, energy-minimized, and simulated for 500 ns in four sets to assess complexes with single, double, triple, and quadruple ligand occupancy. The results revealed progressively increased complex stability as additional ligands bound consecutively, with the quadruple-ligand complex exhibiting the highest stability, demonstrating enhanced binding cooperativity (Fig. 2*B*).

Through molecular dynamics simulations, the LG1b–RNA complex maintained structural stability, whereas LG1a exhibited distortions, reflecting the superior hydrogen-bonding capability of base E in LG1b compared to base e in LG1a. Ligand binding was more favorable with the Watson strand (R1) than the Crick strand (R2) (Fig. 2 *C* and *D*), consistent with the established antiparallel binding preference of γPNA to DNA or RNA (30). Distance analyses confirmed stable hydrogen-bonding interactions (~2 Å) between bases E, F, and K, and their corresponding RNA base-pairs (*SI Appendix*, Fig. S1). These results demonstrate that triplet nucleic acid ligands could be rationally designed to selectively recognize and bind pathogenic rCUG^exp^, potentially distinguishing expanded repeats from wild-type sequences.

### Chemical Synthesis.

The synthesis of carboxymethyl nucleobases (e, E, F, and K) and their corresponding γPNA building blocks has been previously reported (*SI Appendix*, Fig. S2) (31). Ligands were assembled via solid-phase synthesis, then cleaved, deprotected, purified by HPLC, and characterized by MALDI-TOF (*SI Appendix*, Figs. S3 and S4 and Table S1). For this study, ligands LG2a and LG2b (Fig. 1*A*) were selected due to prior evidence showing that ligands targeting terminal mismatched RNA base-pairs exhibit superior binding compared to fully complementary ligands (LG1a and LG1b) (28), likely driven by kinetic factors. Additionally, we synthesized LG2c by adding (*L*-Arg)_6_ to a lysine sidechain containing a disulfide bond to enhance cellular uptake and intracellular (cytoplasmic and nuclear) cargo release, addressing the limited permeability of LG2b (*SI Appendix*, Figs. S5 and S6). UV-vis spectroscopy of the deprotected building blocks (*SI Appendix*, Fig. S7), individually measured in water and a 1:1 mixture of H_2_O/MeOH at ambient temperature, and collectively within ligands in sodium phosphate buffer at 95 °C (*SI Appendix*, Fig. S8), showed consistent agreement. The extinction coefficients measured at 260 nm were K = 20,370 ± 1,475; F = 5,787 ± 391; e = 4,383 ± 200; E = 14,683 ± 895 M^−1^ cm^−1^.

### Assessment of Ligand Binding.

**Fig. 3.**
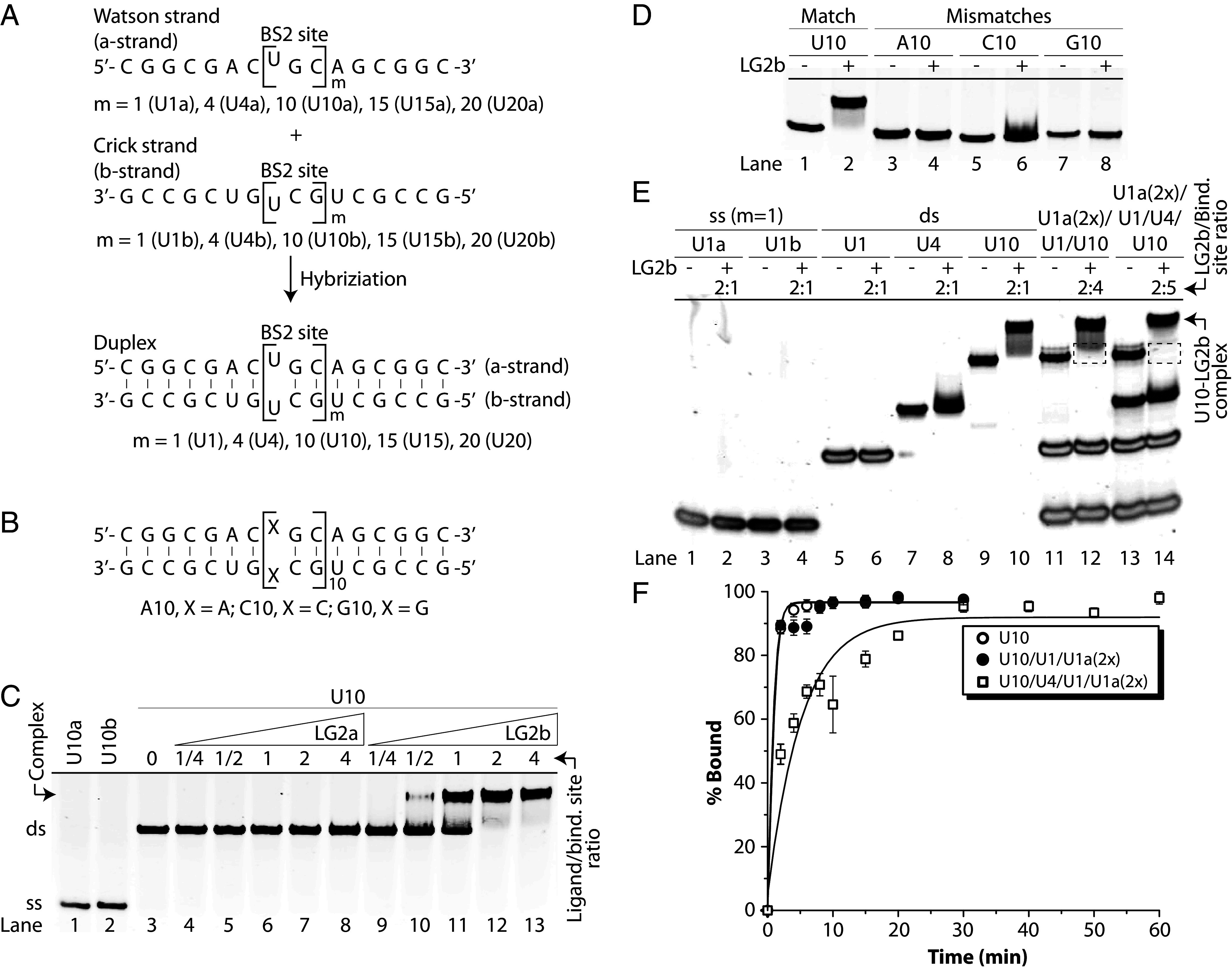
Ligand binding assessment with model RNAs. (*A*) Sequences of model RNA duplexes containing varying numbers of LG2a/b binding sites, formed by hybridizing equimolar Watson and Crick strands. (*B*) Sequences of mismatched RNA duplexes. (*C*) EMSA results comparing LG2a (lanes 4 to 8) and LG2b (lanes 9 to 13) binding to U10. U10 concentration: 500 nM (5 µM ligand-binding sites); ligand-to-binding site ratios indicated. (*D*) LG2b binding comparison between perfectly matched U10 (U<>U, lanes 1 & 2) and mismatched A10 (A<>A, lanes 3 & 4), C10 (C<>C, lanes 5 & 6), and G10 (G<>G, lanes 7 & 8) RNAs. RNA concentration: 500 nM (5 µM ligand-binding sites); LG2b: 10 µM (2:1 ligand-to-binding site ratio). (*E*) LG2b binding analysis with single-stranded (U1a, U1b) and double-stranded (U1, U4, U10) RNAs, individually (lanes 1 to 10) and in combinations (lanes 11 to 14) to assess selectivity. LG2b: 10 µM; RNA concentrations adjusted to maintain a 5 µM binding site concentration (2:1 ligand-to-binding site ratio in lanes 1 to 10). In lanes 11 to 14, U1a and U1b concentrations were doubled to compensate for the absence of a complementary strand. Lanes 11 & 12: U1a (10 µM), U1 (5 µM), U10 (0.5 µM)—20 µM ligand-binding sites. Lanes 13 & 14: Same as lanes 11 & 12, with U4 (1.25 µM) added—25 µM ligand-binding sites. (*F*) Competitive binding over time under the same conditions as lanes 10, 12, and 14 in (*E*). Samples were prepared in physiological ionic strength buffer (10 mM sodium phosphate, 137 mM NaCl, 150 mM KCl, 2 mM MgCl_2_, pH 7.2), incubated at 37 °C for 30 min, separated by 15% nondenaturing PAGE, and stained with SYBR Gold.

Electrophoretic mobility shift assays (EMSAs) were performed to evaluate ligand–RNA binding. RNA substrate U10 (Fig. 3*A*), containing 10 perfectly matched ligand-binding sites, was incubated with varying concentrations of ligands LG2a or LG2b under physiologically relevant conditions (10 mM NaPi, 137 mM NaCl, 150 mM KCl, 2 mM MgCl_2_, pH 7.4, 37 °C) (32), unless stated otherwise. Samples were analyzed using nondenaturing PAGE and visualized with SYBR-Gold staining. As shown in Fig. 3*C*, LG2a displayed negligible binding even at high ligand-to-target ratios (lanes 4 to 8; up to 4:1 ratio, lane 8). In contrast, LG2b exhibited ~60% binding at a 1:1 ratio (lane 11 vs. lane 6), achieving complete binding at a 2:1 ratio, as indicated by the clear formation of shifted complexes and reciprocal disappearance of free RNA bands. The sharply defined, stable shifted bands persisted throughout the 2-h electrophoresis at room temperature, suggesting minimal complex dissociation. These results confirm that LG2b (bearing base E instead of e) binds RNA targets significantly more effectively than LG2a, in agreement with molecular dynamics simulations. Enhanced binding by LG2b likely arises from stronger hydrogen-bonding and more favorable base-stacking interactions, resulting in improved target affinity.

Although the shifted bands indicated ligand–RNA binding, whether this interaction was sequence-specific or driven by nonspecific electrostatic or hydrophobic interactions remained unclear. To address this question, LG2b was incubated with single-base mismatched RNA targets: A10 (A–A), C10 (C–C), and G10 (G–G) (Fig. 3*B*), using a 2:1 ligand-to-binding site ratio sufficient for complete binding to the matched U10 target. No shifted bands were observed with any mismatched sequences (Fig. 3*D*, lanes 3 to 8), confirming the sequence-specific binding of LG2b to U10. This high specificity likely arises from the bifacial nature of the ligand interactions, enabling precise recognition and discrimination of RNA sequences.

### Evaluation of LG2b Binding Selectivity.

A significant challenge in targeting rCUG-repeats is distinguishing pathogenic length from normal-length sequences. To assess the selectivity of LG2b, we performed competitive binding assays using single-stranded RNAs (U1a and U1b) and their corresponding duplexes containing one (U1), four (U4), or ten (U10) binding sites (Fig. 3*A*). This approach allowed us to determine whether LG2b recognizes structured RNA duplexes exclusively or also binds single-stranded RNA. LG2b showed no detectable binding to single-stranded RNA (U1a or U1b; Fig. 3*E*, lanes 1 to 4) or shorter duplexes (U1 and U4; lanes 5 to 8), whereas robust binding was observed with the longer duplex U10 (lanes 9 to 10). Importantly, excess single-stranded U1a and duplexes U1 or U4 did not disrupt LG2b binding to U10 (lanes 11 to 14), further confirming LG2b’s selectivity for longer rCUG-repeat duplexes. Kinetic analysis demonstrated rapid and complete binding to U10 within 5 min (Fig. 3*F*). However, in the presence of excess U4, binding exhibited a lag phase, reaching full occupancy in about 20 min. This indicates weaker and less stable interactions with shorter repeats, allowing rapid dissociation and preferential reassociation with longer, more stable duplexes such as U10. Collectively, these findings highlight LG2b’s strong selectivity for longer rCUG-repeat sequences, underscoring its downstream potential for targeting triplet-repeat expansions.

**Fig. 4.**
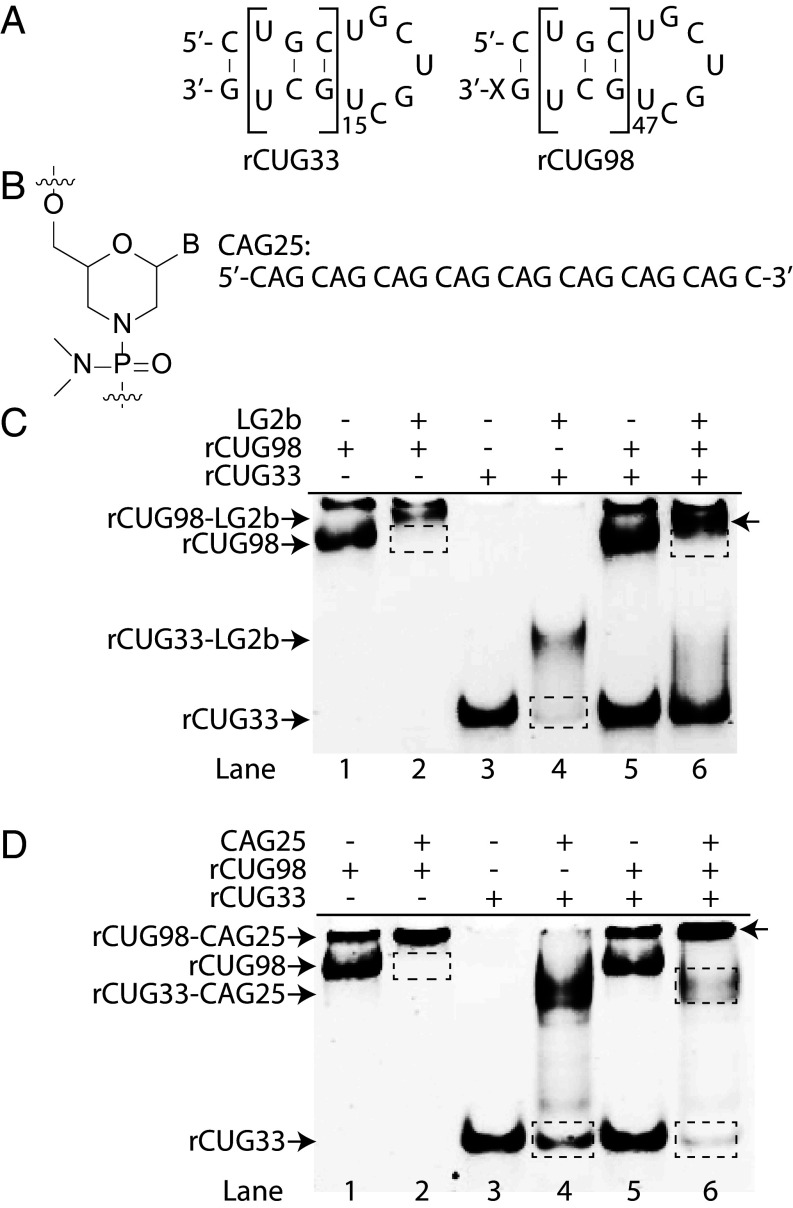
Assessment of ligand binding selectivity. (*A*) RNA targets with normal (rCUG33) and pathogenic (rCUG98) repeat-lengths. (*B*) Sequence of the morpholino oligonucleotide CAG25. (*C*) Evaluation of LG2b’s binding selectivity for rCUG33 and rCUG98, using LG2b (5 µM), rCUG33 (0.33 µM, ~5 µM ligand-binding sites), and rCUG98 (0.10 µM, ~5 µM ligand-binding sites). (*D*) Comparison of LG2b and CAG25 binding selectivity for normal and pathogenic rCUG-repeat lengths. The LG2b, rCUG33, and rCUG98 concentrations matched those in (*C*), while CAG25 (1.2 µM) was used at a stoichiometric ratio with its binding sites in rCUG33 (0.33 µM ~ 1.2 µM) and rCUG98 (0.1 µM ~ 1.2 µM). All samples were prepared under physiologically relevant ionic strength and temperature. The top bands in (*C* and *D*) likely represent large rCUG98 complexes formed through intermolecular hybridization, which could not be resolved by nondenaturing PAGE under the given experimental conditions.

Although LG2b showed promising selectivity, the U10 duplex (10 binding sites) does not represent pathogenic repeat lengths seen in DM1. To determine if LG2b distinguishes pathogenic from normal-length rCUG-repeats, we compared its binding to pathogenic-length rCUG98 (98 repeats, 47 ligand-binding sites) and normal-length rCUG33 (33 repeats, 15 binding sites) duplexes in their native hairpin motif (Fig. 4*A*). rCUG98 was chosen due to its established pathogenic role in DM1 (22). For reference, we included the antisense morpholino oligonucleotide CAG25 (Fig. 4*B*), containing eight CAG-repeats, previously demonstrated to effectively disrupt rCUG^exp^–MBNL1 complexes and correct misspliced mRNAs in DM1 cells and animal models (22). LG2b and CAG25 were incubated separately with equimolar binding sites of rCUG98 and rCUG33 duplexes at a 1:2 ligand-to-binding site ratio. Consistent with earlier observations, LG2b preferentially bound pathogenic-length rCUG98 with no detectable interaction with rCUG33 (Fig. 4*C*, lanes 5 & 6). In contrast, CAG25 showed nearly equal binding to both rCUG98 and rCUG33 under the same conditions (Fig. 4*D*, lanes 5 & 6). The observed preferential binding of LG2b to longer repeats highlights strong cooperativity, likely driven by intermolecular π–π stacking between adjacent ligands, enabled by their extended aromatic surfaces.

### Quantification and Validation of Ligand Binding.

We determined the dissociation constants (K_d_s) of LG2b for rCUG-repeated RNAs of varying lengths using EMSAs. Initial experiments provided approximate K_d_ values, which were subsequently refined through precise measurements across a 100-fold ligand concentration range centered around these initial estimates (*SI Appendix*, Fig. S9). As a positive control, we included the CAG25 morpholino oligonucleotide, whose affinity (K_d_) for rCUG98 is well established (22). All EMSAs were performed in triplicate using 15% PAGE, and RNA–ligand (or RNA-CAG25) complexes were visualized by SYBR-Gold staining. The fraction of RNA bound at each LG2b concentration was quantified using ImageJ, and resulting data were plotted and fitted to a Hill–Langmuir binding model using Origin software to determine K_d_ values.

**Fig. 5.**
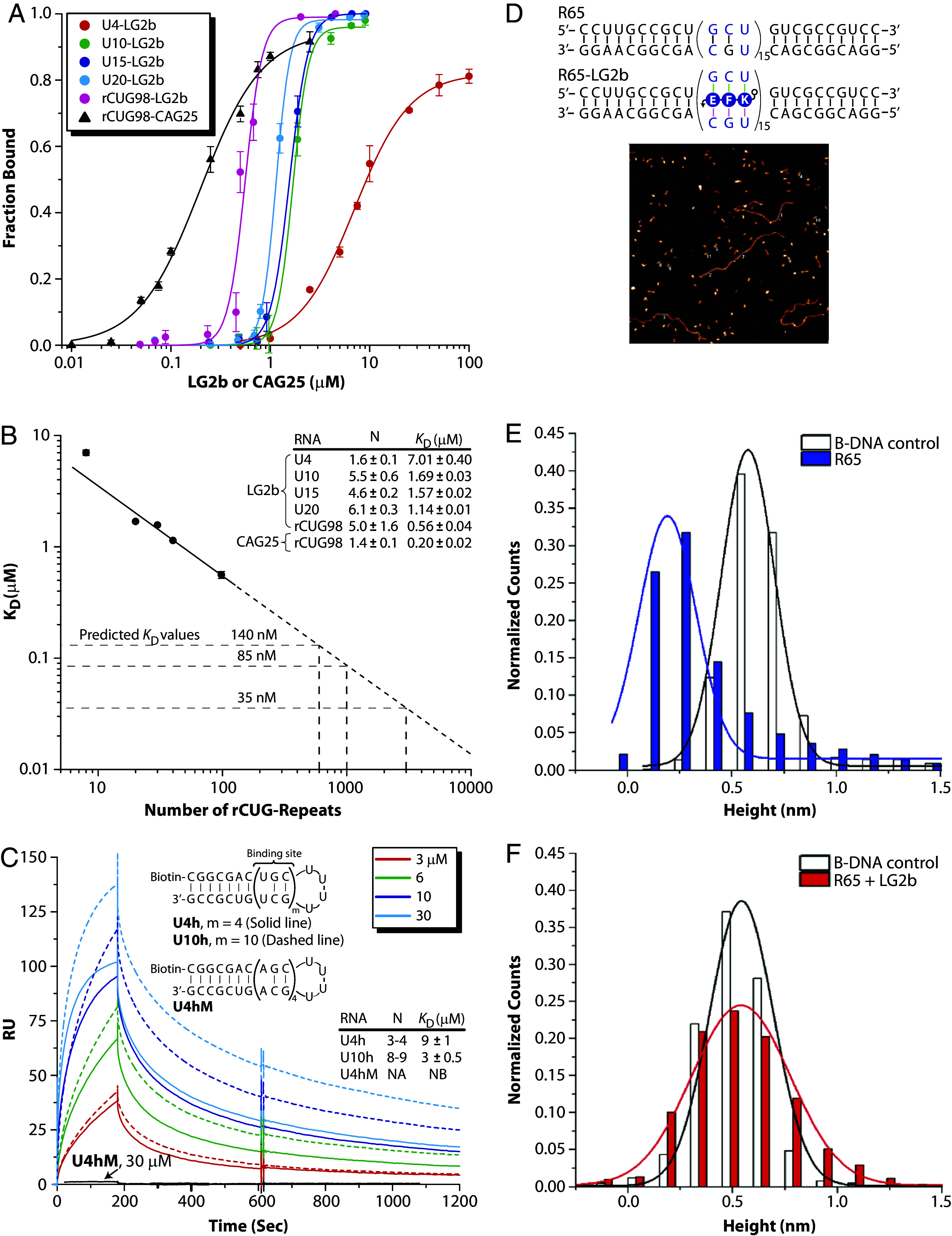
Quantification of LG2b binding. (*A*) Hill plots showing LG2b binding to U4, U10, U15, U20, and rCUG98, along with the binding of CAG25 to rCUG98 for comparison, as determined by EMSA. (*B*) Correlation between K_d_ values and rCUG-repeat lengths. Filled circles indicate experimental data, the solid line represents the linear regression fit, and the dashed line shows extrapolation. (*C*) SPR profiles of LG2b binding to U4h (4 sites, line), U10h (10 sites, dashes), and mismatched U4hM. (*D*) Sequence of R65 without and with bound LG2b, alongside an AFM image of R65–LG2b complexes (oval shapes) and reference linear 1,434-bp B-DNA (string-like feature). (*E*) Histogram comparing the height distribution of R65 and reference DNA. (*F*) Histogram comparing the height distribution of R65–LG2b complexes and reference DNA. Gaussian fits indicate heights of R65 (0.191 ± 0.011 nm), R65 + LG2b (0.540 ± 0.007 nm), and B-DNA (0.560 ± 0.003 nm), with a 0.348 nm increase upon complex formation.

As expected, the K_d_ values for LG2b decreased progressively with increasing rCUG-repeat length, reflecting enhanced binding affinity and greater cooperativity (Fig. 5*A*). This trend was further supported by an increase in the Hill coefficient (N), rising from ~1.6 for shorter repeats (U4) to ~5 for pathogenic-length rCUG98. Specifically, LG2b exhibited K_d_ values of 7.01 ± 0.4, 1.69 ± 0.03, 1.57 ± 0.02, 1.14 ± 0.01, and 0.56 ± 0.04 µM for U4, U10, U15, U20, and rCUG98, respectively. In comparison, CAG25 bound rCUG98 with a K_d_ of 0.20 ± 0.02 µM, closely matching its previously reported affinity (232 ± 47 nM) (22). Although CAG25 displayed slightly higher affinity (~twofold lower K_d_) for rCUG98, its binding cooperativity was significantly lower (N = 1.4) compared to LG2b (N ~ 5) (Fig. 5*B*). This difference is evident from the broader binding profile of CAG25 compared to the sharper and more cooperative binding curves observed for LG2b (Fig. 5*A*).

In neuromuscular disorders involving triplet-repeat expansions, selectively targeting pathogenic RNA transcripts without affecting normal lengths is crucial, as many implicated genes play essential roles in normal development (33). Nonspecific targeting risks disrupting their normal functions, causing harmful side effects. DM1 is unique among such disorders due to the extreme expansion of rCUG-repeats, often exceeding 1,000 repeats, compared to typical expansions of 50 to 200 repeats in other diseases. LG2b’s bifacial design specifically addresses this challenge by preferentially binding longer pathogenic repeats with enhanced selectivity. Additionally, the observed linear correlation between LG2b’s K_d_ values and rCUG-repeat length predicts a K_d_ of approximately 35 nM for transcripts with 5,000 repeats (Fig. 5*B*)—an affinity comparable to FDA-approved small molecules targeting structured protein sites (34). Overall, these results underscore the potential of this ligand class as molecular tools for studying RNA-repeat expansion pathology and as candidates for therapeutic development.

To validate EMSA results, we employed surface plasmon resonance (SPR) to determine K_d_ values for representative RNA targets. We selected three RNA hairpins—U4h (four binding sites), U10h (ten binding sites), and a single-base mismatched control (U4hM)—designed to facilitate purification and efficient surface immobilization. Each RNA target was 5′-biotinylated and individually immobilized on streptavidin-coated flow cells. SPR experiments were performed at 25 °C in physiologically relevant buffer, with varying concentrations of LG2b injected over immobilized RNAs at a constant flow rate of 100 μL/min, followed by a washing step to observe ligand dissociation. Reference signals from a blank flow cell were subtracted to yield response units (RU) directly proportional to the bound ligand.

SPR sensograms for LG2b binding to U4h and U10h are shown in Fig. 5*C*. Although both targets exhibited similar association rates (on-rates), the dissociation rate (off-rate) was approximately three times slower for U10h compared to U4h, resulting in K_d_ values of 3 µM for U10h and 9 µM for U4h, closely matching EMSA results. Under identical conditions, LG2b showed no detectable binding to the mismatched control (U4hM), confirming the specificity observed by EMSA. Notably, SPR-derived Hill coefficients (N) were approximately twice as high as those obtained by EMSA, reflecting differences in experimental conditions between the two methods. Nonetheless, both techniques consistently demonstrated increased binding cooperativity with repeat length, reinforcing the robustness of these observed trends.

### Evaluation of Ligand Binding by Atomic Force Microscopy (AFM).

High-resolution AFM imaging has previously been used to reveal structural details of nucleic acid complexes, including DNA G-quadruplexes (35). To independently verify LG2b binding, we analyzed the structural topology of a model RNA target (R65) using AFM. R65 consists of 15 (rGCU/rUGC)-repeats—near the upper limit of chemical synthesis—and, consistent with prior designs, includes sufficiently long CG-rich flanking regions to ensure duplex stability (Fig. 5*D*). Binding experiments were conducted under the same conditions as EMSA (*SI Appendix*, Fig. S10), and RNA–ligand complexes were immobilized on a mica surface for imaging. Contour maps generated by AFM allowed visualization of ligand-induced structural changes, using a double-stranded B-DNA of known length as an internal control. The LG2b-bound R65 complex was expected to show increased contour height compared to unbound R65 due to ligand insertion expanding the RNA helix. Since RNA molecules align along their helical axis on mica, changes in contour height directly reflect alterations in helix width, providing structural confirmation of LG2b binding.

Measured contour heights were 0.191 ± 0.011 nm for unbound R65, 0.540 ± 0.007 nm for R65–LG2b complexes, and 0.560 ± 0.003 nm for the control B-DNA (Fig. 5 *E* and *F*). The consistent contour height of B-DNA, regardless of LG2b presence, confirmed the ligand’s lack of interaction with double-stranded DNA. The significant increase in contour height (0.348 nm) observed exclusively for R65 upon LG2b binding strongly suggests that ligand interaction occurred via a pothole-filling mechanism. Unlike typical groove-binding interactions with small molecules or proteins (36)—which generally produce minimal height changes—this substantial height differential provides robust structural evidence of specific ligand–RNA binding. These AFM findings support the sequence-specificity and selective targeting of rCUG-repeated RNAs by LG2b.

### Displacement and Prevention of MBNL1 Binding by LG2b.

**Fig. 6.**
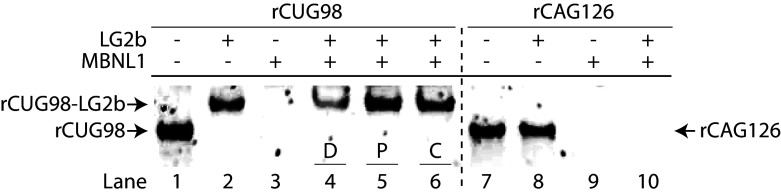
Evaluation of LG2b’s ability to disrupt the CUG98–MBNL1 complex. Displacement, prevention, and competitive binding assays assessing LG2b’s effect on the rCUG98–MBNL1 interaction under different conditions (D: displacement, P: prevention, C: competitive). rCUG98, MBNL1, and LG2b concentrations were 0.01 µM (0.5 µM ligand binding sites), 0.50 µM, and 2.50 µM, respectively.

A primary driver of DM1 pathology is gain-of-function RNA toxicity, wherein expanded CUG-repeat transcripts form hairpin structures that sequester RNA splicing regulators, particularly MBNL1. This sequestration leads to nuclear retention of the MBNL1–rCUG^exp^ complexes, reduced DMPK protein production, and disrupted MBNL1 activity, resulting in widespread RNA-splicing defects characteristic of DM1. To determine whether LG2b can compete effectively with MBNL1 for binding to rCUG98, we conducted competitive binding assays in three distinct formats: 1) *displacement*—LG2b added to preformed rCUG98–MBNL1 complexes; 2) *prevention*—LG2b preincubated with rCUG98 before adding MBNL1; and 3) *competitive*—all components added simultaneously. In the absence of MBNL1, the rCUG98-LG2b complex formed a discrete, strongly shifted band (Fig. 6, lanes 1 & 2). Conversely, the rCUG98–MBNL1 complex appeared as a diffuse smear without a clearly defined band (lane 3), indicative of heterogeneous binding sites—a pattern consistent with previous reports (22). A similar pattern was observed with the negative control rCAG126, indicating that while MBNL1 bound to rCAG126 (lane 9), it was not displaced by LG2b (lane 10), which does not bind rCAG126 (lane 7 vs. lane 8).

In all three competitive binding formats—displacement (lane 4), prevention (lane 5), and competitive (lane 6)—a distinct shifted band consistent with the rCUG98–LG2b complex was observed (lane 2 vs. lane 1). Adding LG2b to the (MBNL1 + rCUG98) sample restored the rCUG98–LG2b band (lane 4 vs. lane 3). This demonstrates LG2b’s ability to effectively disrupt MBNL1 binding, whether MBNL1 was prebound (displacement) or introduced subsequently (prevention). Notably, in the displacement assay, the intensity of the rCUG98–LG2b complex band was approximately threefold lower compared to the prevention and competitive assays (lane 4 vs. lanes 5 and 6), indicating that displacement of prebound MBNL1 by LG2b occurs less efficiently. This observation aligns with previous findings by Zimmerman (37) for small-molecule ligands, where the IC_50_ for displacement (560 nM) was nearly two orders of magnitude higher than for prevention (3 nM), reinforcing that preemptive ligand binding is significantly more effective in inhibiting MBNL1–RNA interactions.

### Restoration of RNA Missplicing in DM1 Patient-Derived Myotubes.

**Fig. 7.**
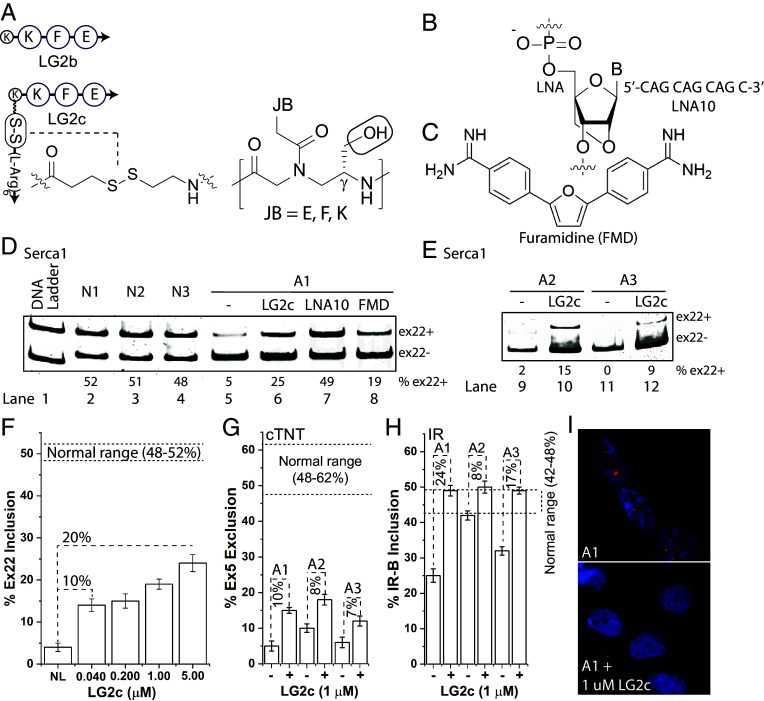
Assessment of LG2c’s ability to restore RNA missplicing. (*A*) LG2c shares LG2b’s recognition elements but features a C-terminal hexa-arginine domain linked via a disulfide bridge to enhance cellular uptake and promote intracellular cargo release. It also featured a γ-hydroxymethyl sidechain in place of a miniPEG, designed to improve RNA binding. (*B* and *C*) Show the chemical structures of the positive controls: locked nucleic acid (LNA) and small-molecule furamidine (FMD), respectively. (*D*) ESMA results showing exon 22 (ex22+) inclusion in Serca1 mRNA from DM1 patient-derived myotubes (A1) treated with 1 µM LG2c, compared to LNA (0.125 µM) and FMD (1 µM), and (normal) unaffected controls (N1, N2, N3). (*E*) Treatments of additional DM1 patient-derived myotubes with LG2c (1 µM). (*F*) Concentration-dependent effects of LG2c on ex22+ in Serca1 mRNA of A1 myotubes. (*G* and *H*) Effects of LG2c (1 µM) on exon 5 (ex5-) exclusion in cTNT and the inclusion of IR-B isoform of IR mRNA, respectively. (*I*) Fluorescent detection of nuclear foci of DM1 patient-derived myotubes (A1) with and without treatment with 1 µM LG2c. Blue: nuclei, red: nuclear foci.

To determine whether the ligand could rescue DM1-associated RNA missplicing, we developed LG2c, an LG2b analog incorporating a disulfide-linked *C*-terminal hexa-arginine domain to enhance cellular uptake and facilitate intracellular cargo release (38). Additionally, the γ-miniPEG sidechain was replaced with a hydroxymethyl group to improve RNA binding by reducing steric hindrance (Fig. 7*A*). DM1 patient-derived myotubes were treated with LG2c alongside positive controls (*SI Appendix*, Figs. S11 and S12), locked nucleic acid (LNA, Fig. 7*B*) (39) and small-molecule furamidine (FMD, Fig. 7*C*) (19), followed by total RNA extraction, cDNA synthesis, and PCR analysis of three genes: Serca1 (sarcoplasmic/endoplasmic reticulum calcium ATPase 1), cTNT (cardiac troponin T), and IR (insulin receptor), chosen for their known splicing defects and associations with DM1 symptoms. Serca1 is linked to delayed muscle relaxation (40), cTNT to cardiac conduction defects and arrhythmia (2), and IR to insulin resistance (41). Fig. 7 *D*–*H* present EMSA results comparing LG2c-treated myotubes with positive controls and unaffected cells, while Fig. 7*I* shows representative nuclear staining of treated and untreated cells.

Inspection of Fig. 7*D* reveals that at 1 µM, LG2c restored inclusion of Serca1 exon 22 (ex22+) in affected myotubes A1 by approximately 20%, compared to 44% for LNA (125 nM) and 14% for FMD (1 µM). While LG2c’s effect is comparable to FMD, it is notably weaker than LNA. However, unlike LNA, which required a delivery vehicle (Lipofectamine 2000), both LG2c and FMD were effective without one, potentially offering advantages for downstream applications. Similar trends were observed in two additional DM1 myotube lines, A2 and A3 (Fig. 7*E*), *albeit* with slightly reduced responses. LG2c’s splicing correction was shown to be concentration-dependent, increasing from ~ 10% at 40 nM to ~ 20% at 5 µM (Fig. 7*F* and *SI Appendix*, Fig. S13). Comparable improvements were observed in the exclusion of exon 5 (ex5-) of cTNT (Fig. 7*G* and *SI Appendix*, Fig. S14) and the inclusion of IR-B isoform of IR (Fig. 7*H* and *SI Appendix*, Fig. S15), with the latter showing a more pronounced effect. Supporting these findings, nuclear staining revealed a marked reduction in nuclear foci following treatment with 1 µM LG2c compared to untreated cells, with representative images shown in Fig. 7*I*. Notably, the reduction in nuclear foci appears more substantial than the extent of splicing restoration, though the reason for this discrepancy remains unclear. Nonetheless, the data consistently demonstrate that LG2c improves splicing defects in DM1 myotubes, with efficiency comparable to that of small-molecule FMD.

## Conclusions

This study marks a significant step forward in the rational design of RNA targeting molecules, showcasing how short nucleic acid ligands can achieve high specificity through bifacial recognition. While challenges remain, particularly in enhancing cellular uptake and achieving full functional rescue, the ability to selectively target pathogenic RNA repeats without affecting normal transcripts highlights the downstream therapeutic and diagnostic potentials of this approach. Such a molecular platform is not only suitable for rCUG-repeats or related RNA triplet-repeat expansions but also holds promise for targeting a diverse array of native RNA structures with essential physiological roles.

## Materials and Methods

### Monomers and Oligomers.

The synthesis of γPNA monomers with Janus bases E, F, and K have been reported (31); and oligomer synthesis is detailed in the Supporting Information.

### Molecular Dynamics Simulations of the LG1 Series Ligands.

The eKF and EKF triads were constructed using Chimera and optimized with the *HF/6-31G* basis set in Gaussian. The RNA–ligand complex (RNA-LIG-RNA) was built using the NAB module and incorporated these triads. A modified MeγPNA backbone from crystal structure 3PA0 was grafted onto the helical RNA framework, forming a 12-base pair triple helix. This structure underwent energy minimization via the steepest descent method, was solvated with TIP3P water and ionized to maintain physiological conditions. After a second energy minimization, the system was gradually heated to 300 K with a 25 kcal/mol/Å position restraint on the RNA–ligand complex. Six short simulations progressively released these restraints, culminating in an unrestrained 500 ns NPT simulation at 300 K and 1 bar using a Nose–Hoover thermostat and a Parrinello–Rahman barostat. All MD simulations were performed in GROMACS with a 2 fs time step. Electrostatic interactions were calculated using the particle mesh Ewald method with a 10 Å cutoff. The Amber99-parmbsc0 force field with χOL3 modifications was used for RNA, while ligand parameters were derived from RED-generated amber atom types and charges.

### RNA Sample Preparation.

RNA targets with varying rCUG-repeat lengths and CG-rich terminal regions were selected, with the latter to enhance duplex stability under physiological conditions (Fig. 3*A*). Both perfect-match (U<>U) and mismatch variants (A<>A, C<>C, G<>G) were examined (Fig. 3*B*). Intermolecular duplexes, formed by hybridizing two complementary RNA strands, were used instead of hairpins to allow more flexible target design and to compare ligand binding to single-stranded RNA vs. r(CUG/GUC)_n_-duplexes. All samples were prepared in physiological relevant buffer (10 mM NaPi, 137 mM NaCl, 150 mM KCl, 2 mM MgCl_2_, and pH7) and incubated with ligands at 37 °C.

### Preparation of rCUG98 and rCAG126 Transcripts.

Following established protocols (22), rCUG98 and rCAG126 transcripts were enzymatically synthesized using T7 RNA polymerase with plasmids pCUGexp-tail and pCAGexp-tail as templates. The plasmids were linearized with *XbaI* (rCUG98) or *SacI* (rCAG126) digestion at 37 °C for 2 h, separated on a 1% agarose gel, and purified using the QIAquick Gel Extraction Kit. Transcription was performed in a 40 µL reaction containing 2 μg of digested plasmid DNA, 3.5 mM rNTPs, 4 µL enzymatic mix (T7 RNA polymerase with RNase inhibitors, NEB), and 1× reaction buffer. The reaction proceeded at 37 °C for 4 h, followed by annealing at 70 °C for 5 min and rapid cooling to 0 °C. Transcripts were purified via 10% nondenaturing PAGE, visualized by UV-shadowing, excised, and extracted into 1× PBS buffer.

### Electrophoretic Mobility-Shift Assay (EMSA).

RNA and RNA–ligand complexes were separated on 8 to 15% nondenaturing PAGE (1× TBE, 10 V/cm), stained with SYBR-Gold, and imaged. Band intensities were quantified using ImageJ. For K_D_ determination, binding assays were performed at constant RNA concentration with varying ligand levels, and fraction bound vs. log[ligand] data were fitted using a Hill binding model.

### Surface Plasmon Resonance (SPR).

SPR measurements were performed with a four-channel Biacore T200 optical biosensor system. Flow cell 1 was left blank, while flow cells 2 to 4 were immobilized with 5′-biotin-labeled RNA sequences. The SPR experiments were performed at 25 °C in degassed and filtered 1× PBS, 2 mM MgCl_2_, 150 mM KCl; pH 7.4. Solutions of different known LG2b concentrations were injected over the immobilized RNA surface at a flow rate of 100 μL/min. Compound solution flow was then replaced by buffer flow resulting in dissociation of the complex. After each cycle, the sensor chip surface was regenerated with 1M KCl as a regeneration buffer for 60 s followed by three running buffer injections (each 60 s) to yield unbound RNA and a stable baseline for the following cycles. The reference response from the blank cell was subtracted from the response in each flow cell containing RNA to give a signal (RU, response units) that is directly proportional to the amount of bound compound.

### Atomic Force Microscopy (AFM).

#### Sample preparation.

Each sample contained 30 ng of R65/R65–LG2b, 45 ng of double-stranded DNA, and excess water, adjusted to a total volume of 20 µL. A freshly cleaved mica surface was rinsed with deionized water, blow-dried with compressed air, and treated with 20 µL of 16.7 µM spermidine. The spermidine was then blow-dried and rinsed with deionized water. A drop of 20 µL of the sample was deposited on the mica surface. After an incubation time of 5 min, the mica surface was blow-dried using compressed air, rinsed with deionized water and dried once again before AFM imaging.

#### Imaging.

All AFM images were acquired using a Cypher ES SPM (Asylum Research, Oxford Instruments) in AC Air Topography mode at 25 °C. Scanasyst-fluid+ probes (Bruker Corp., USA) with a stiffness of 0.7 N/m were used. A pulsed blue laser (BlueDrive) with a ~5 µm circular spot size was focused on the cantilever base for photothermal excitation, while a superluminescent diode (SLD) laser (3 × 9 µm spot size) was used for detecting cantilever deflections.

#### Image analysis.

Image analysis was performed using custom software developed in MATLAB and CellProfiler, with histograms generated in OriginLab. Height images were extracted from raw IGOR Pro binary wave files (.ibw) and converted into 16-bit intensity images for processing. Using CellProfiler’s “EnhanceOrSuppressFeatures” module, long, thin features (Control B-DNA) and spot-like features (RNA particles) were enhanced. Particles were identified based on intensity thresholds, determined using the Global Otsu method, which minimizes variance within foreground and background pixel classes. Control B-DNA and RNA particles were distinguished via size and shape filtering. Identified particles were then converted into image masks and overlaid onto the original height image in MATLAB. Overlapping pixels were extracted and plotted as height histograms in OriginLab. Finally, CellProfiler’s “MeasureObjectShapeSize”module was used to measure the major and minor axis lengths of particles. The aspect ratio (major/minor axis) was calculated and plotted as a histogram in OriginLab.

### Competitive Binding of LG2b and MBNL1 with rCUG98.

Samples were prepared in a physiologically relevant buffer with 0.1 µg/µL BSA and incubated at 37 °C. Binding conditions: D (Displacement)—rCUG98 and MBNL1 were preincubated, followed by LG2b addition; P (Prevention)—CUG98 and LG2b were pre-incubated, followed by MBNL1 addition; Competitive—all three components were incubated together. rCUG98, MBNL1, and LG2b concentrations were 0.01 µM (0.5 µM ligand binding sites), 0.50 µM, and 2.50 µM, respectively. Samples were resolved on an 8% nondenaturing PAGE gel (15 V/cm, 3 h) and stained with SYBR-Gold.

### Cell Culture.

Fibroblasts derived from DM1 patients (cell lines A1: GM03132, A2: GM03759 and A3: GM03989 expressing DMPK transcript with ∼2,000 CTG-repeats) and control fibroblasts derived from non-DM1 patients (cell lines N1: GM03377, N2: GM03651, N3: GM03652) were purchased from the Coriell Cell Repositories. Cells were grown in Dulbecco’s modified Eagle’s Medium (DMEM) (Thermo Fisher Scientific) supplemented with 10% fetal bovine serum (FBS) (Thermo Fisher Scientific), 1% antibiotic antimycotic solution (Sigma-Aldrich), and 1% nonessential amino acids solution (Thermo Fisher Scientific), in 5% CO_2_ atmosphere at 37 °C. At the differentiation stage, MyoD-transduced fibroblasts were cultured in a differentiation medium containing DMEM/F-12 (HAM) 1:1 (Thermo Fisher Scientific), 2% donor equine serum (DES) (Cytiva), and 1% antibiotic antimycotic solution. The medium was changed every 2 to 3 d.

### MyoD Transduction.

The Adenovirus MyoD was obtained from Vector Biolabs with an infectivity of 1 × 10^7^ MOI/µL. The fibroblasts at 30% confluence grown in normal growth medium were transduced with 2% MyoD (20 MOI/cell). Seventy-two hours after incubation in 5% CO_2_ atmosphere at 37 °C, the virus-containing transduction medium was replaced with fresh differentiation medium. The MyoD-transduced fibroblasts were cultured for 10 d to differentiate into myotubes.

### Treatments.

LG2c (40 nM to 5 µM) and Furamidine (1 µM) were incubated with myotubes after 10 d of differentiation. Cell transfection experiments were performed using Lipofectamine 2000 (Invitrogen) according to the manufacturer’s instructions with PO-LNA-CAG-10 at 125 nM final concentration. The total RNA was isolated from cells after 72 h of treatment, using RNeasy (Qiagen) with in-column gDNA removal according to the manufacturer’s protocol and the RNA concentrations were determined using NanoDrop (Thermo Fisher Scientific).

### PCR Reactions.

First-strand cDNA was synthesized using 1 µg of total RNA using QuantiTect Reverse Transcription Kit (Qiagen) according to the manufacturer’s instructions and subsequent PCR was performed using 2 µL of the cDNA. All PCRs were performed at an initial holding temperature of 95 °C for 3 min, 30 cycles of denaturation at 95 °C for 30 s, annealing at 50 to 60 °C for 30 s, and elongation at 72 °C for 30 s, and a final elongation temperature of 72 °C for 10 min and 4 °C holding temperature. Primer sequences and PCR conditions are specified in *SI Appendix*, Table S2. The RT-PCR products were separated in 5% nondenaturing PAGE (15 V/cm, 1.5 h) and stained with SYBR-Gold. The amounts of PCR products were quantified by ImageJ.

### Nuclear Staining.

Myotubes were treated with 1 µM LG2c for 3 d, fixed with 4% paraformaldehyde, permeabilized with 70% EtOH, hybridized with Cy3-(CAG)_8_ probe, and imaged on an Olympus-IX80 with a 100×-oil objective. Blue: nuclei, red: nuclear foci.

## Supplementary Material

Appendix 01 (PDF)

## Data Availability

Study data are included in the article and/or *SI Appendix*.
