# Supplementary material for: A pothole-filling strategy for selective targeting of rCUG-repeats associated with myotonic dystrophy type 1
Source: Proc Natl Acad Sci U S A. 2026 Jan 9;123(2):e2507065123. doi: 10.1073/pnas.2507065123 (PMC12799113; doi:10.1073/pnas.2507065123)
Supplement: Supplementary file 1 — Appendix 01 (PDF) [file pnas.2507065123.sapp.pdf]

SUPPLEMENTAL INFORMATION

A Pothole-Filling Strategy for Selective Targeting of rCUG-Repeats Associated with Myotonic Dystrophy Type 1

J. Dinithi R. Perera,[a†] Shivaji A. Thadke,[a†] Savani W. Thrikawala,[a†] Isha Dhami,[a] V. M. Hridya,[b] Arnab Mukherjee,[b] Ananya Paul,[c] W. David Wilson,[c] Keith W. R. Tan,[d] Nicholas Z. W. Chan,[d] Anh Tuân Phan,[d] and Danith H. Ly[a*]

[a] Department of Chemistry and Institute for Biomolecular Design and Discovery (IBD), Carnegie Mellon University, Pittsburgh, PA 15213. [b] Department of Chemistry, Indian Institute of Science Education and Research (IISER), Pune, Maharashtra 411008, India. [c] Department of Chemistry, Georgia State University, Atlanta, GA 30302. [d] School of Physical and Mathematical Sciences, Nanyang Technological University, Singapore.

[†] These authors contributed equally.

[*] To whom correspondence should be addressed: dly@andrew.cmu.edu

Table of Contents Page

Table S1	Sequence compositions of ligands	3
Table S2	Primers employed in PCR reactions	3
Fig. S1	Hydrogen-bonding interaction of Janus bases with RNA base pairs	4
Fig. S2	Structures of chemical building blocks	5
Fig. S3	HPLC profiles of LG2a and LG2b	6
Fig. S4	MALDI-TOF spectra of LG2a and LG2b	7
Fig. S5	HPLC profile of LG2c	8
Fig. S6	MALDI-TOF spectrum of LG2c	9
Fig. S7	UV-vis spectra of K, F, e, and E	10
Fig. S8	UV-vis spectra of LG2a and LG2b	11
Fig. S9	EMSA data of LG2b binding with U10 used in K_d determination	12
Fig. S10	EMSA data of LG2b binding with R65	13
Fig. S11	DIC images of myotubes	14
Fig. S12	Myoblasts and myotubes derived from GM03132 fibroblasts	15
Fig. S13	Concentration dependent rescue of Serca1 by LG2c	16
Fig. S14	RNA splicing rescue of cTNT by LG2c	17
Fig. S15	RNA splicing rescue of IR by LG2c	18

Procedure for solid-phase ligand synthesis 19-20

Table S1. Sequence compositions of ligands

**Ligands**	**Sequence**
LG1b	H-FKE-k-NH_2
LG2a	H-eFK-k-NH_2
LG2b	H-EFK-k-NH_2
LG2c	H-EFK-x-r_6-k-NH_2

k= *L*-lysine, r= *L*-arginine, x= 3-((2-aminoethyl)disulfaneyl)propanoic acid. Oligomer sequences are written from N- to C-terminus. LG1b, LG2a, and LG2b contained charge-neutral γ-MiniPEG backbone; while LG2c contained γ-hydroxymethyl backbone.

Table S2. Primers employed in PCR reactions

Transcript	Forward Primer (5'-3')	Reverse Primer (5'-3')	Annealing temperature	# of cycles	Fragment sizes
Serca1 *ex22*	GCTCATGGTCCTC AAGATCTCAC	AGCTCTGCCTGA AGATGTGTCAC	50 °C	30	204/162
IR *ex11*	CCAAAGACAGACT CTCAGAT	AACATCGCCAAG GGACCTGC	60 °C	30	168/135
cTNT *ex5*	ATAGAAGAGGTGG TGGAAGAGTAC	GTCTCAGCCTCT GCTTCAGCATCC	60 °C	30	134/104

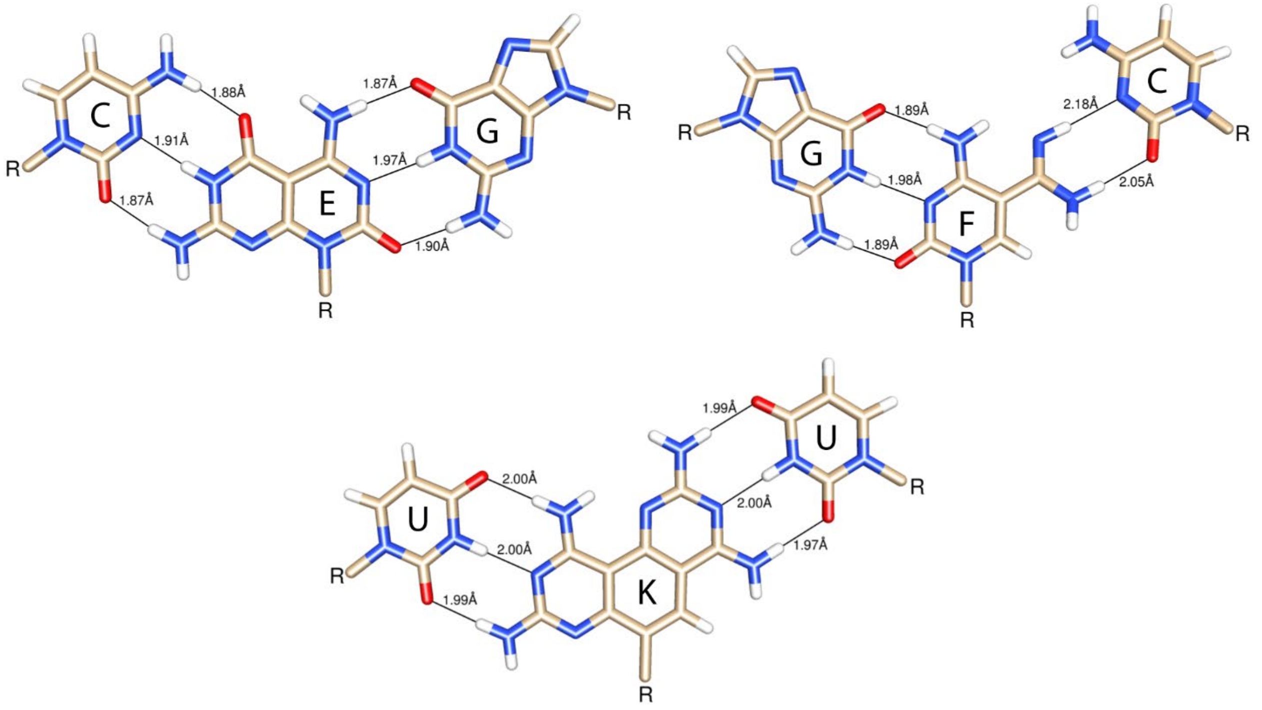

Fig. S1. Hydrogen-bonding interaction of Janus bases E, F, and K with their respective RNA base-pairs.

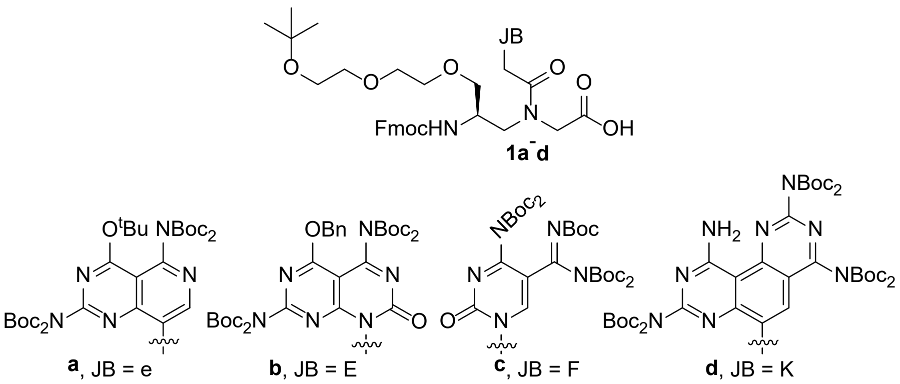

Fig. S2. Structures of chemical building blocks.

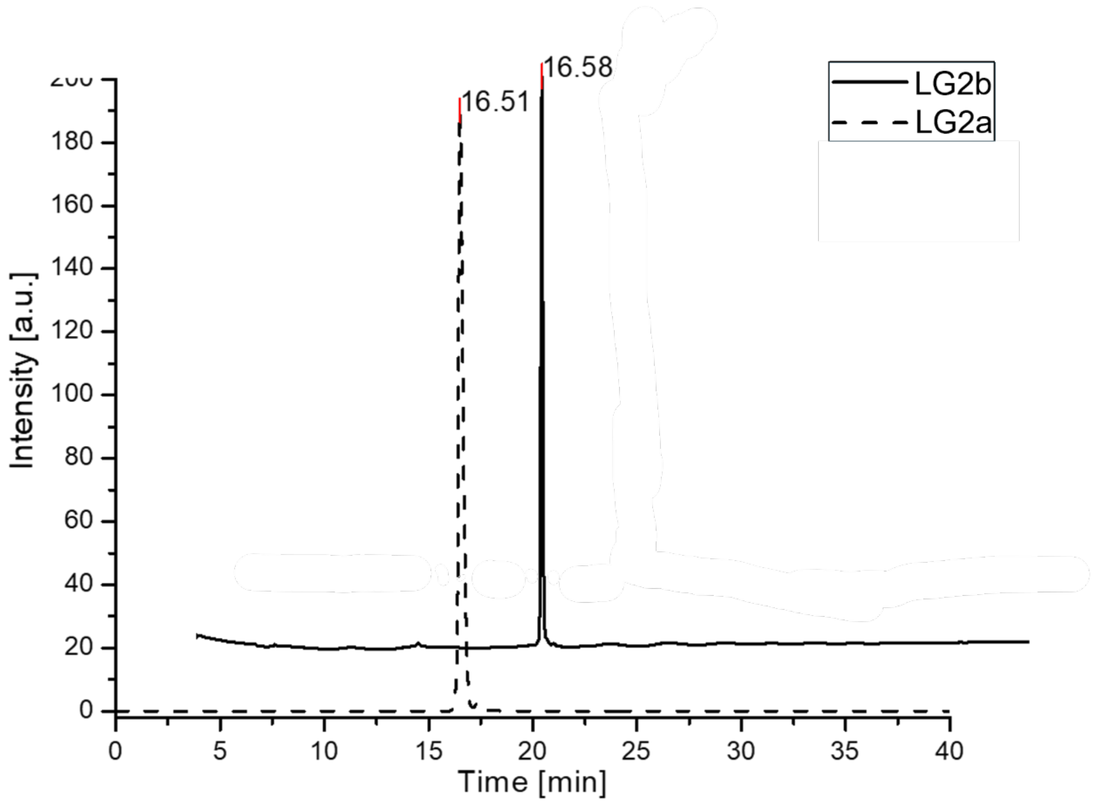

Fig. S3. HPLC profiles of LG2a (16.51 min, dashed line) and LG2b (16.58 min, solid line). HPLC condition: mobile phase A: water (0.1%TFA), mobile phase B: Acetonitrile (0.1%TFA). Gradient: 5-40% B over 40 min at 55 °C, wavelength: 260 nm.

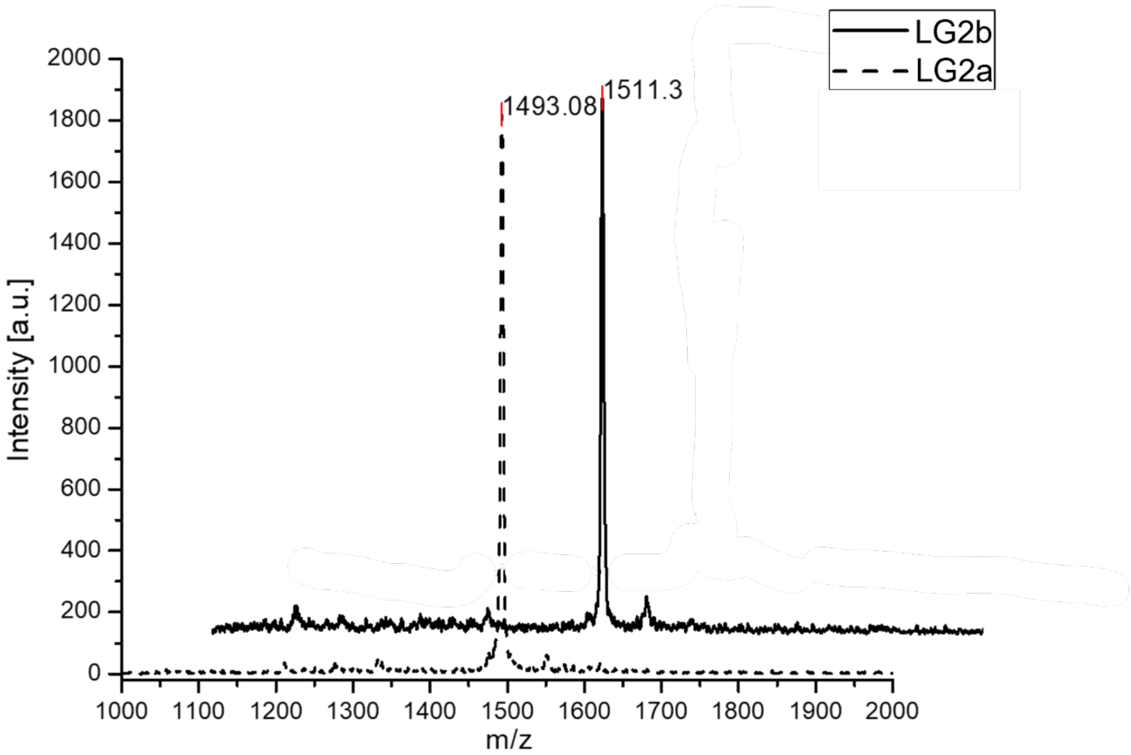

Fig. S4. MALDI-TOF spectra of LG2a (dashed line, calculated m/z: 1491.72, observed m/z: 1493.08) and LG2b (solid line, calculated m/z: 1507.71, observed m/z: 1511.30).

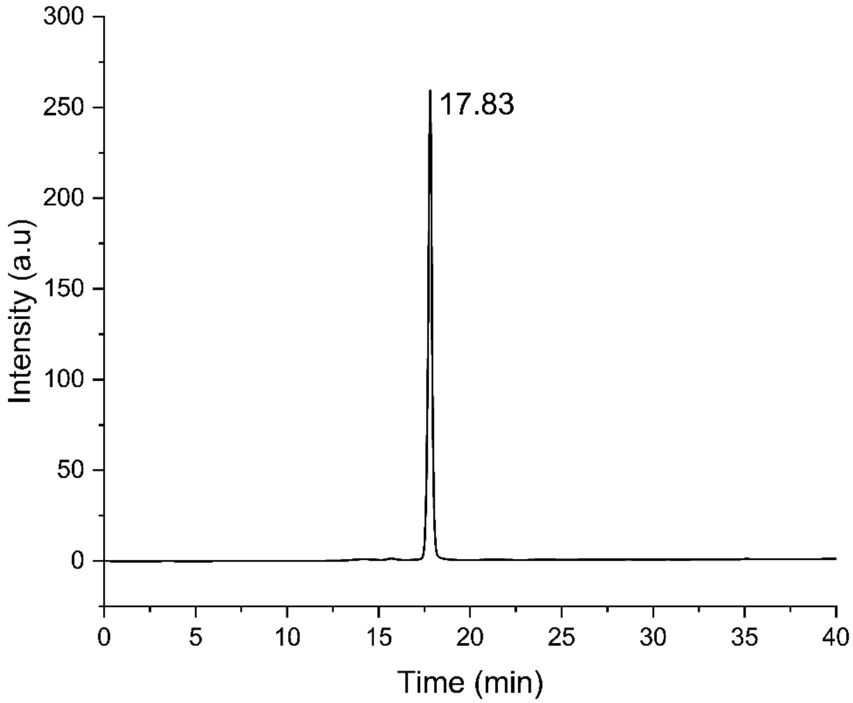

Fig. S5. HPLC profile of LG2c (17.83 min at 260 nm). HPLC condition: mobile phase A: Water (0.1%TFA), mobile phase B: acetonitrile (0.1%TFA). Gradient: 10-80% B over 40 min at 55 °C.

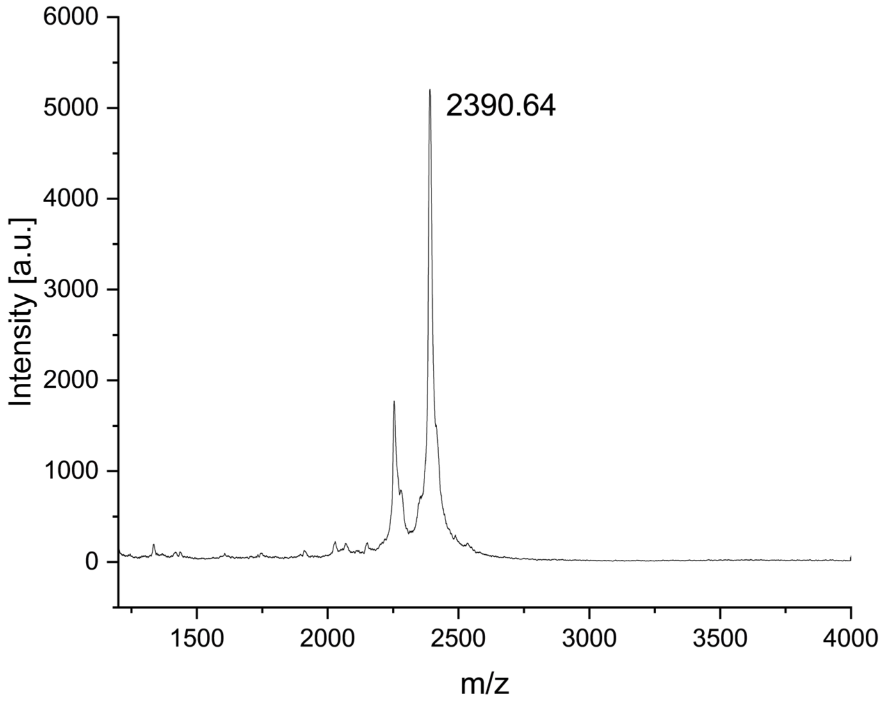

Fig. S6. MALDI-TOF spectrum of LG2c (calculated m/z: 2352.23 observed m/z: 2390.64).

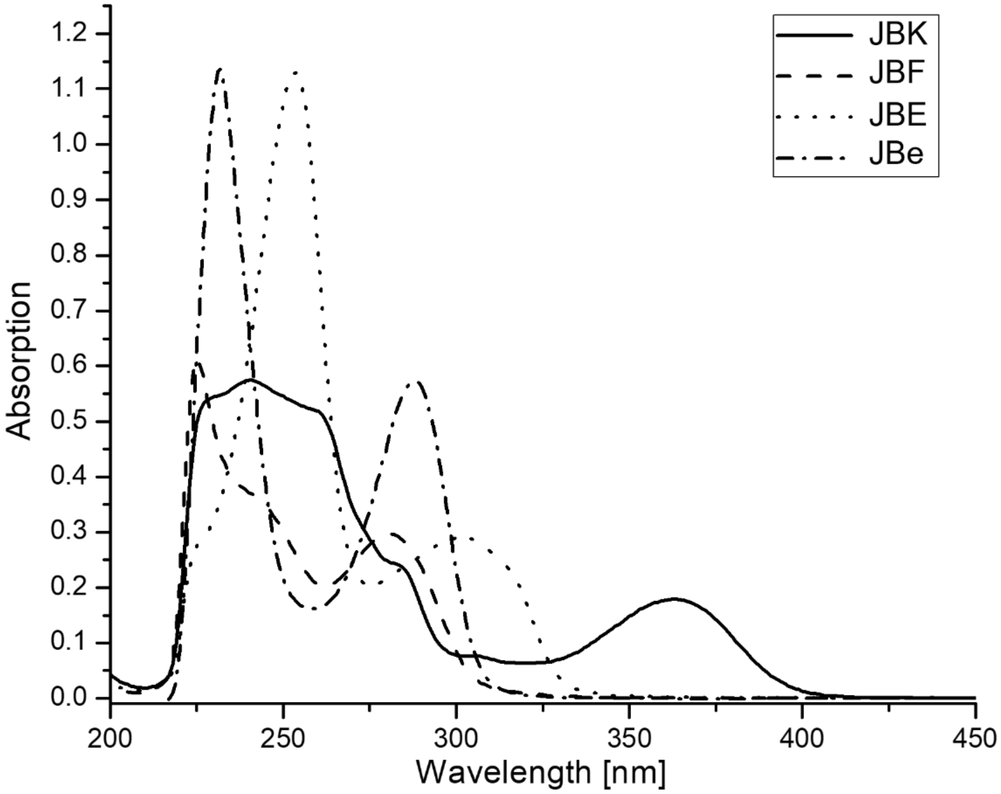

Fig. S7. UV-vis spectra of K, F, e, and E. *Sample preparation:* 5 mg of K (Boc_6) acetic acid, F (Boc_5) acetic acid, e (Boc_4) acetic acid, and 5.5 mg of E (Boc_4) acetic acid were treated with 50% TFA/DCM for 1 h. Volatiles were removed via vacuum centrifugation at room temperature for 2 h. The resulting crude products were dissolved in 10% DMSO/water (1 mL). From this stock, 5%, 10%, 15%, 20%, and 25% dilutions were prepared in water. *UV-vis measurements:* 10, 5, 3.34, 2.50, and 2 μL of each solution were diluted to 1 mL with water. UV-vis absorption was measured at room temperature and 95°C (no spectral pattern changes observed). The baseline of the same solution was subtracted from each absorption profile. *Extinction coefficient determinations:* The UV-Vis absorptions at 260 nm for K, F, e, and E were 0.498, 0.203, 0.554, and 0.157, respectively. All extinction coefficients were calculated based on five averaged UV-vis absorption readings at 260 nm of known concentration by using Beer-Lambert law (A = ecl, A= Absorption, c = concentration, l= optical path length = 1, e = extinction co-efficient or absorptivity). The extinction coefficients: K = 20370 ± 1475, F = 5787 ± 391, E = 14683 ± 895, e = 4383 ± 200.

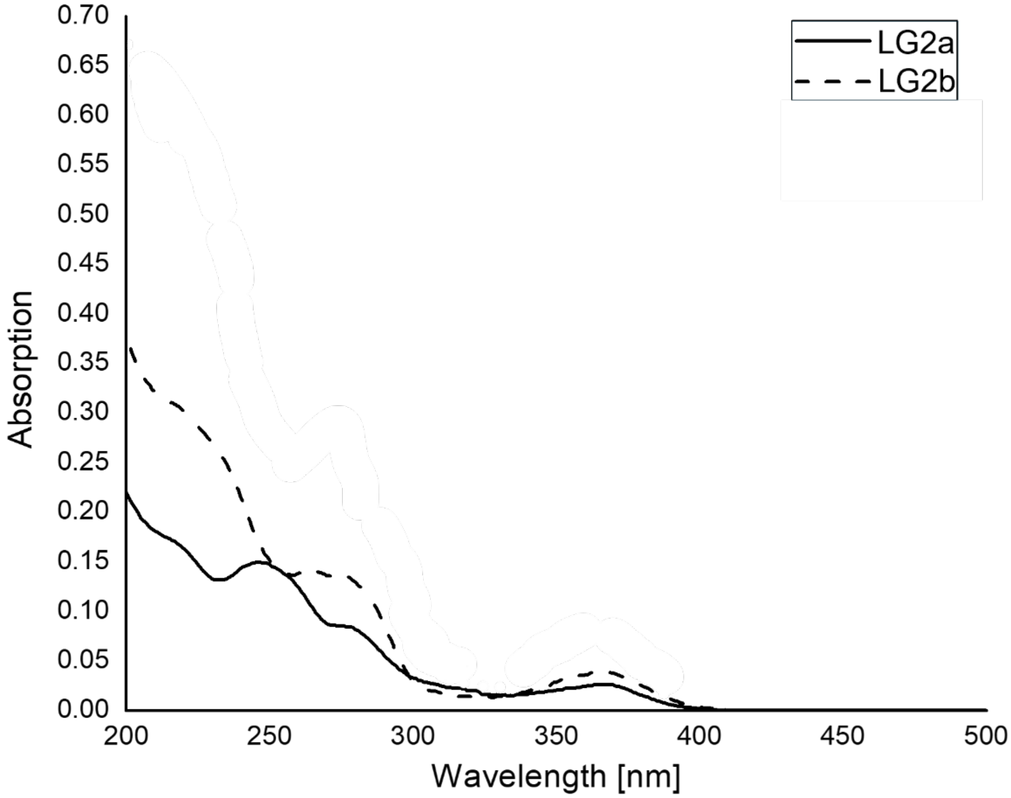

Fig. S8. UV-vis spectra of LG2a (solid line) and LG2b (dashed line) at 95 °C. The extinction coefficients (ε) for LG2a and LG2b used in this study were 30,540 and 40,840 $M^{-1}cm^{-1}$, respectively.

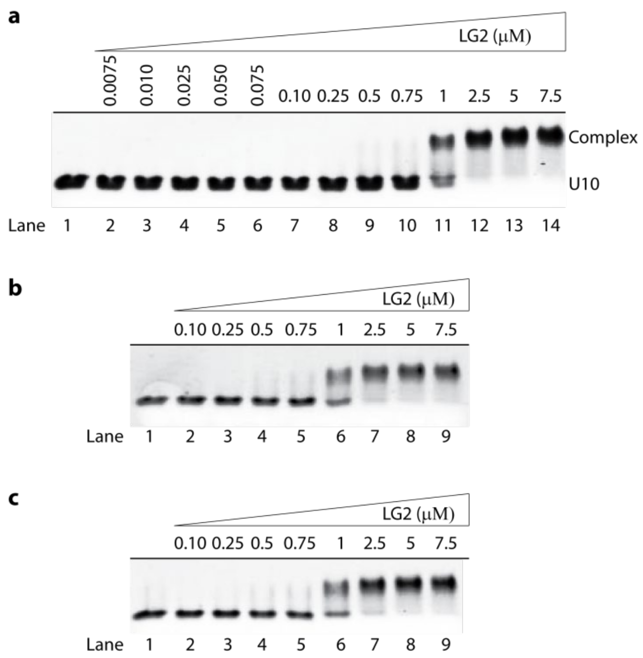

Fig. S9. Example of EMSA data of LG2b binding with U10 used in K_d determination. The concentration of U10 was 100 nM, and those of LG2 are as shown. The samples were prepared in physically relevant buffer and incubated at 37 °C for 1 h prior to separation by 15%-non-denaturing PAGE and stained with SYBR-Gold. The fraction bound was quantified by Image J. Panels **a**-**c** are the triplicate experiments used to determine the K_d value of LG2b with U10.

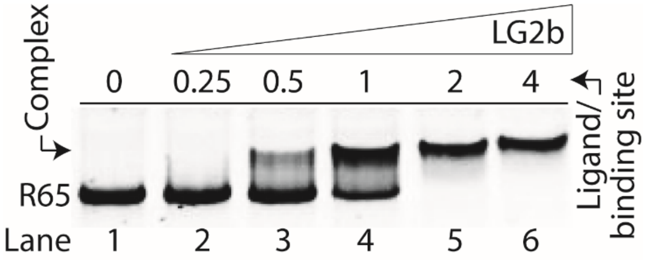

Fig. S10. EMSA data of LG2b binding with R65. The R65 duplex concentration was 330 nM (corresponding to 3.3 μM ligand binding sites), while LG2b concentrations in lanes 2 through 6 were 1.25, 2.5, 5, 10, and 20 μM, respectively. Samples were prepared in a buffer mimicking physiological ionic strength (10 mM NaPi, 137 mM NaCl, 150 mM KCl, 2 mM $MgCl_2$, pH 7.4) and incubated at 37°C for 1 h before separation. Non-denaturing 15% TBE-PAGE was performed at 12.5 V/cm for 1.5 hours to analyze the binding interactions.

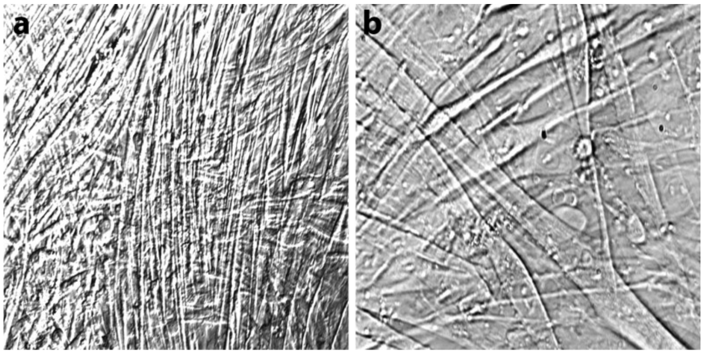

Fig. S11. DIC images of myotubes at (**a**) 10-X and (**b**) 40-X magnification. Fibroblasts were differentiated to myoblasts by transduction with Ad-MyoD for 3 d, followed by myotube induction by culturing cells in DMEM/F-12 50/50 medium containing 2% (vol.) donor equine serum for 7 d. Images were taken by Olympus-IX81 microscope.

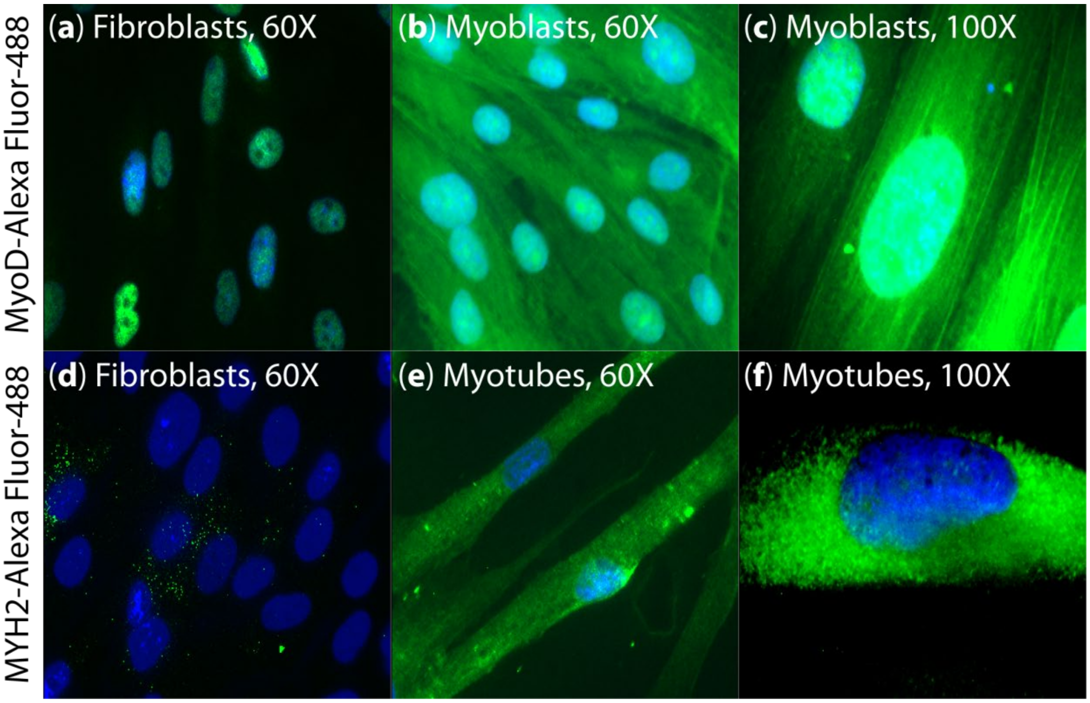

Fig. S12. Myoblasts and myotubes derived from GM03132 fibroblasts stained with primary antibody-conjugates, MyoD-Alexa Fluor-488 and MYH2-Alexa Fluor-488, and with DAPI. (**a**,**d**) Fibroblasts, (**b**,**c**) myoblasts, and (**e**,**f**) myotubes. Images were acquired on an Olympus-IX81 fluorescence microscope with the indicated oil-objectives (60X and 100X).

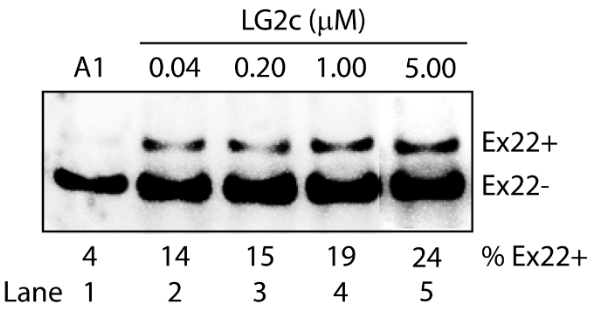

Fig. S13. Concentration dependent splicing rescue of Serca1 by LG2c following 72-h treatment. A1: affected DM1 myotubes.

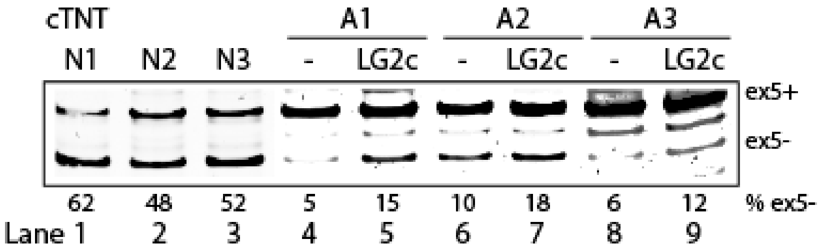

Fig. S14. RNA splicing rescue of cTNT by LG2c at 1 μM concentration following 72-h treatment. N1, N2, N3: unaffected; and A1, A2, A3: affected DM1 myotubes.

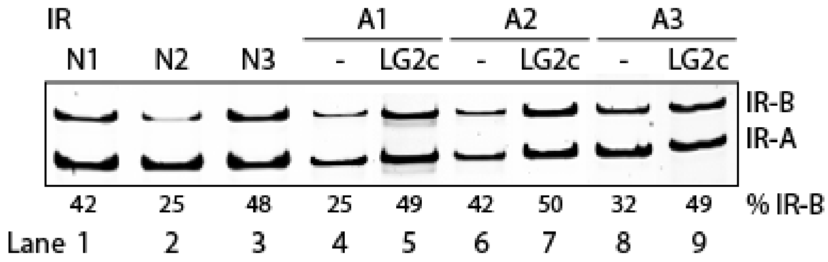

Fig. S15. RNA splicing rescue of IR by LG2c at 1 μM concentration following 72-h treatment. N1, N2, N3: unaffected; and A1, A2, A3: affected DM1 myotubes.

Solid-Phase Ligand Synthesis

Resin loading [(MP)(PAL)]

(1) MiniPEG loading. 1 g of MBHA resin (1 mmol/g, Peptide International, RMB-2100-PI) was soaked in DCM for 1 h, then washed with DCM (3×) and 5% DIEA in DCM (3×). The Kaiser test confirmed neutralized amine groups (blue). In a 15-mL tube, 3.5 mL NMP, 450 µL **Solution A**, **460 µL Solution B**, and **550 µL Solution C** were sequentially added, vortexed for 10 sec, and left to stand for 3 min before transferring to the resin. The reaction proceeded for 1 h with gentle shaking. After removing the reaction mixture under positive air pressure, the resin was washed with DMF (3×), DCM (3×), 5% DIEA in DCM (1×), and DCM (3×). For capping, the **Capping Solution** was added, and the resin was gently agitated for 45 min (2×). The final wash with DCM (3×) was followed by Kaiser test confirmation (pale yellow), indicating completion.

Solution A (0.200 M Fmoc-MP in NMP): 43 mg Fmoc-MP dissolved in 500 µL NMP
Solution B (0.500 M DIEA in Pyridine): 87 µL DIEA mixed with 913 µL pyridine
Solution C (0.201 M HATU in NMP): 55 mg HATU dissolved in 750 µL NMP
Capping Solution (Ac_2O/NMP/pyridine:1/2/2): 2 mL Ac_2O, 4 mL NMP, and 4 mL pyridine

(2) PAL and Lys loading (1 g of resin from above)

Fmoc was removed by treating the resin with 20% piperidine in DMF (2×, 7 min each), followed by washes with DMF (3×) and DCM (3×). Successful deprotection was confirmed *via* Kaiser test (blue). For coupling, the following solutions were mixed and activated for 3 min before addition to the resin:

3 mL of 0.2 M **Monomer Solution**
1.5 mL of 0.52 M **DIEA Solution**
1.5 mL of 0.39 M **HBTU Solution**

The reaction proceeded for 30 min with gentle agitation. Completion was confirmed by Kaiser test (pale yellow). The resin was then washed with DMF (3×), 5% DIEA in DMF (1×), and DCM (1×). To cap unreacted amines, **Capping Solution** was added, and the resin was gently agitated for 30 min. After removing the **Capping Solution**, the resin was washed sequentially with 20% piperidine (2×), DMF (2×), and DCM (2×).

Solution Preparations

Monomer Solution (0.200 M in NMP): 303 mg Fmoc-PAL in 3 mL NMP
281 mg Fmoc-Lys(Boc)-OH in 3 mL NMP
DIEA Solution (0.52 M in DMF): 364 µL DIEA in 3.638 mL DMF
HBTU Solution (0.39 M in DMF): 740 mg HBTU in 5 mL DMF
Capping Solution (Ac_2O/NMP/Pyridine, 1:25:25 vol.)

Fmoc Deprotection

The Fmoc group was removed by treating 50 mg resin with 0.5 mL of 20% piperidine in DMF (2×, 7 min each), followed by washes with DMF (3×) and DCM (3×). Deprotection was confirmed by Kaiser test (blue).

Monomer Coupling

After confirming Fmoc removal, the resin was washed with DMF (4×) and DCM (5×). The coupling solution was prepared by mixing:

150 µL of 0.2 M **Monomer Solution**

75 µL of 0.52 M **DIEA Solution**

75 µL of 0.39 M **HBTU Solution**

The mixture was activated for 10 min, while the resin was washed with pyridine (1×). The monomer solution was then added to the resin, and the reaction proceeded for 2–4 hours with gentle agitation. Completion was confirmed by Kaiser test (pale yellow). The resin was washed with DMF (3×) and DCM (3×).

Capping

Unreacted amines were capped using freshly prepared **Capping Solution** (1:25:25 Ac_2O:NMP:Pyridine). The resin was agitated for 4 min, then washed with DMF (4×) and DCM (5×).

Cleavage

After the final Fmoc deprotection, the resin was washed with DMF (5×) and DCM (8×), then dried under vacuum for 15 min. Cleavage was performed using freshly prepared 95% TFA / 5% m-cresol (0.6 mL per 50 mg resin) at room temperature for 1 hour. The TFA solution was collected in a centrifuge tube, and an additional 0.5 mL cleavage solution was added to the resin for 30 min. The two TFA solutions were combined.

Precipitation

Cold, dry diethyl ether (-60°C, 14 mL) was added to the collected TFA solution and shaken. Precipitation occurred within 30 min. The precipitated oligomer was collected by centrifugation and washed with cold diethyl ether (2×).

Purification

The crude oligomer was dissolved in 0.5 mL of 95% water, 5% acetonitrile, and 0.1% TFA, then purified using reverse-phase analytical chromatography.
